# Endoscopic management of pancreaticopleural fistula in a pediatric patient: A case report and literature review

**DOI:** 10.1097/MD.0000000000020657

**Published:** 2020-06-05

**Authors:** Jing Yang, Lei Lu, Hang-bin Jin, Jian-feng Yang, Xiao-feng Zhang

**Affiliations:** Department of Gastroenterology, Affiliated Hangzhou First People's Hospital, Zhejiang University School of Medicine, Hangzhou, Zhejiang Province, China.

**Keywords:** diagnosis, endoscopic intervention, pancreatic fistula, pediatric patient, surgery

## Abstract

**Introduction:**

Pancreaticopleural fistula (PPF) is a rare but serious complication of pancreatic disorders. As the clinical presentations of PPF are often deceptive, it can cause a delay in the timely diagnosis and proper treatment. PPF is extremely uncommon in pediatric patients, and diagnostic and management strategies for PPF among pediatric patients are scanty.

**Patient concerns:**

A 12-year-old girl presented with cough and dyspnea owing to massive right-side pleural effusion confirmed by Chest X-ray. Biochemical examination of pleural effusion revealed a significant elevation of amylase level. Imaging modalities showed dilated pancreatic duct and fistulous tract connecting pancreatic duct and right thorax.

**Diagnosis:**

Chronic pancreatitis with PPF was diagnosed.

**Interventions:**

Medical therapy was initially attempted for 2 weeks. Endoscopic therapy with naso-pancreatic drainage tube placement was then performed without any complications after failed medical therapy.

**Outcomes:**

The patient has remained healthy and symptom-free during 2 years of follow-up.

**Conclusion:**

When pediatric patients presented with recurrent pleural effusion with unknown etiology, PPF should be taken into consideration. Pleural effusion amylase level is the most important laboratory test and magnetic resonance cholangiopancreatography is recommended to visualize the fistula. Optimal management of PPF should be based on pancreatic duct morphology.

## Abstract 2

Supplemental Digital Content is available in the text

## 1 Introduction

Pancreaticopleural fistula (PPF) is a rare, confusing entity and typically presents as recurrent, massive, blood-stained pleural effusion.^[1]^ It can been observed in patients with acute and chronic pancreatitis or pancreatic trauma.^[2]^ As the clinical presentations of PPF is often deceptive, the diagnosis can be missed, which will cause a delay in proper treatment. PPF is extremely uncommon in pediatric patients, and diagnostic and management strategies of PPF for pediatric patients are scanty. We herein describe a pediatric patient with PPF, and review the relevant reports since 1976 to 2018 to provide a systematic review of the current views on PPF, discussing its presentation and evaluation method, and offering practical advice on its management.

## 2 Case presentation

**Figure 1:**
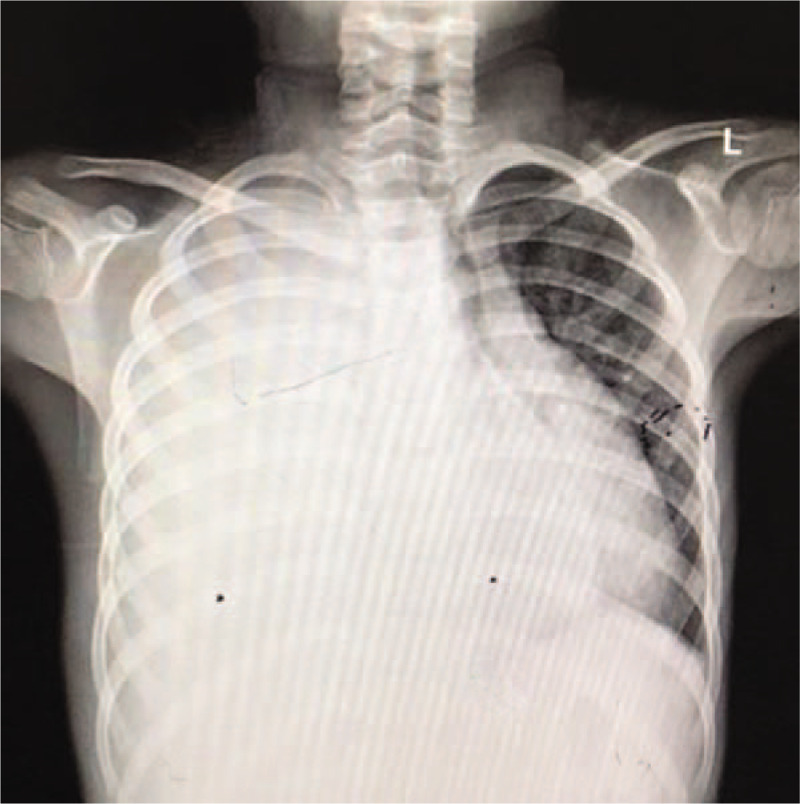
Chest X-ray showed massive pleural effusion in the right thorax.

**Figure 2:**
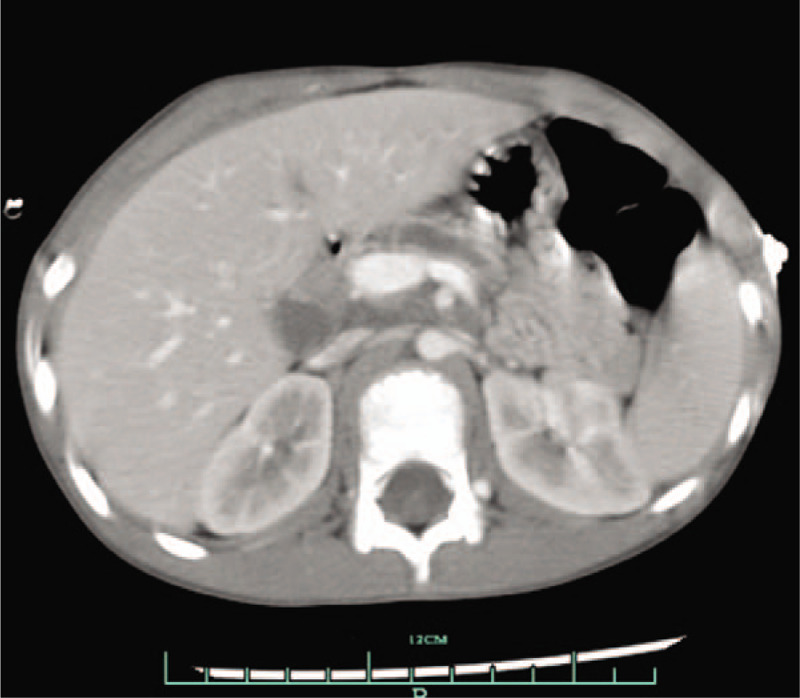
Abdominal CT showed dilated irregular pancreatic duct. CT = computed tomography.

**Figure 3:**
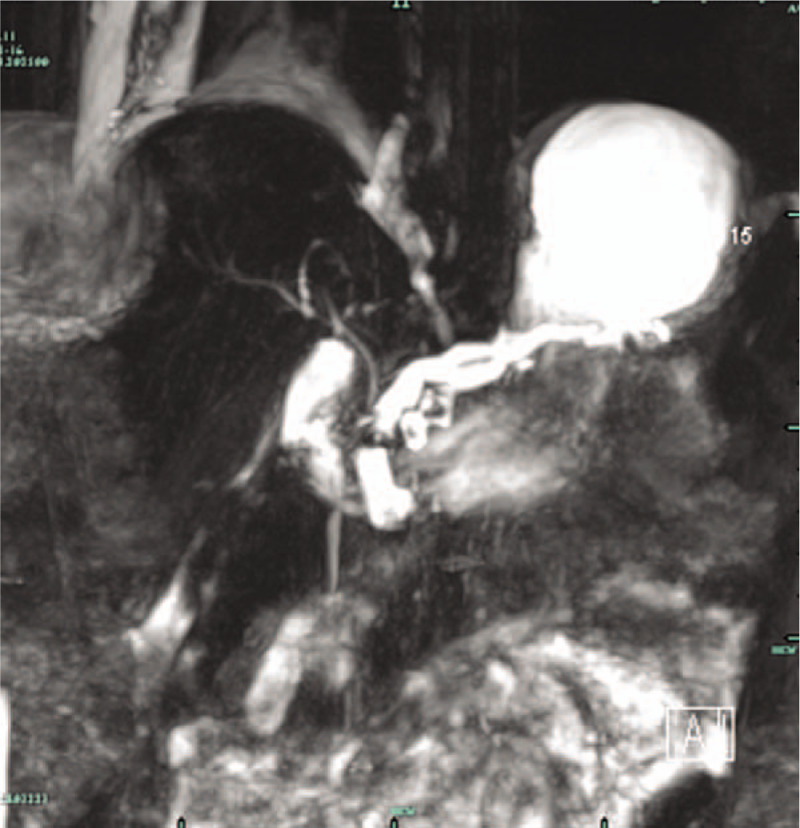
MRCP showed dilated pancreatic duct and a fistulous tract originating from pancreatic duct and extended to the right thorax. MRCP = magnetic resonance cholangiopancreatography.

A 12-year-old girl was admitted to our hospital with cough and dyspnea for 1 month. She also complained of sporadic epigastric pain during the last year. She denied history of abdominal trauma. Physical examinations revealed tachypnea, decreased breath sounds and dull percussion note on the right thorax. The rest of physical examination was unremarkable. Laboratory data showed a mild elevation of serum amylase (504.8 U/L) and lipase (134 U/L). Routine blood tests, serum calcium, serum lipid profile and blood glucose were within normal limits. Chest X-ray showed massive pleural effusion in the right thorax (Fig. 1). A chest tube was then inserted, releasing blood-stained pleural fluid, which resulted in marked clinical improvement. Biochemical examinations of pleural fluid revealed a significant elevation of amylase level of 56,365.7 U/L, and a total protein of 27.6 g/L. No specific pathology was detected, including tuberculosis, connective tissue diseases, rheumatic diseases, or malignancy. Abdominal computed tomography (CT) showed dilated irregular pancreatic duct (Fig. 2). Magnetic resonance cholangiopancreatography (MRCP) showed dilated pancreatic duct, consistent with chronic pancreatitis, together with a fistulous tract originating from pancreatic duct and subsequently extending to the right thorax (Fig. 3). On the basis of medical history, radiological examinations, massive pleural effusion, and elevation of pleural effusion amylase level, a diagnosis of chronic pancreatitis with PPF was considered.

**Figure 4:**
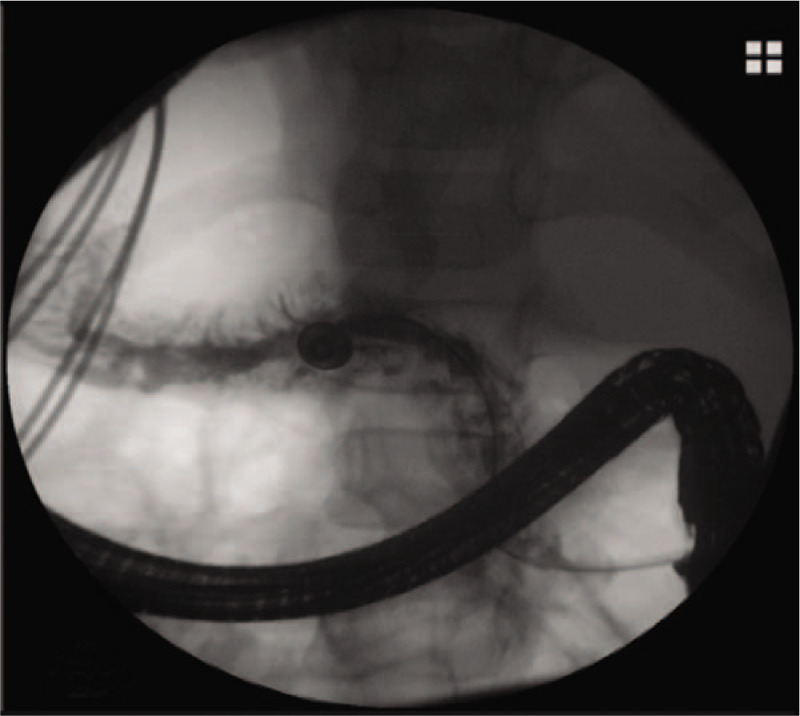
ERCP showed a dilated main pancreatic duct with multiple filling defect. ERCP = endoscopic retrograde cholangiopancreatography.

**Figure 5:**
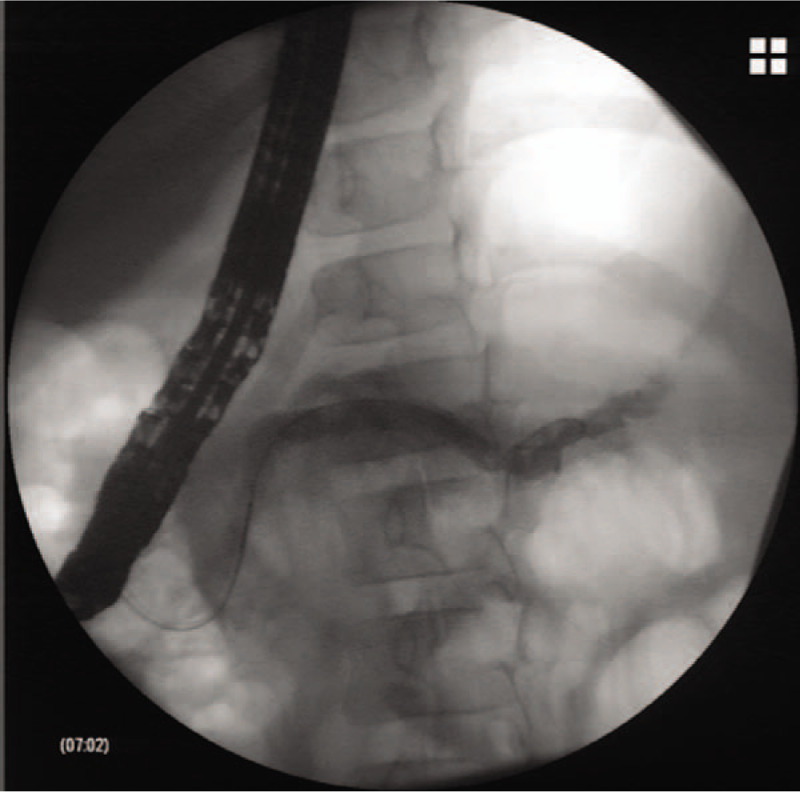
Repeated pancreatogram showed amelioration of the dilated pancreatic duct.

The girl was then treated conservatively with fasting, omeprazole, somatostatin, antibiotic, total parenteral nutrition and chest tube drainage. During the next 2 weeks, the daily drainage volume from chest tube varied from 100 ml to 200 mL. Endoscopic intervention was therefore advised. After obtaining informed consent from the patient’ family, we performed endoscopic retrograde cholangiopancreatography (ERCP), which showed a dilated main pancreatic duct, together with multiple filling defect, an obvious fistula was not identified (Fig. 4). Minor papilla cannulation was failed. Following endoscopic sphincterotomy, a 7 Fr naso-pancreatic drainage tube (NPD) was inserted into the main pancreatic duct. After NPD placement, the chest tube drainage volume was obviously decreased, approximately 20 mL blood-stained fluid from the NPD daily. The NPD was then cut in the duodenum converting to internal drainage 5 days after ERCP. The patient made an uneventful recovery and was discharged from the hospital 2 days later. Four months later, the patient returned for retrieval of the pancreatic stent without any complaints. The dilation of pancreatic duct was relieved, as confirmed by pancreatogram (Fig. 5), and stent is no longer needed. The patient has remained healthy and symptom-free during 2 years of follow-up. The key information of the patient's history is summarized in supplementary timeline picture. Written informed consent was obtained from the patient's guardian for reporting the case details. Because this article does not involve any human or animal trials, it did not require institutional ethical review and approval.

## 3 Literature review

### 3.1 Data sources and search strategy

A comprehensive review of literature was performed on the MEDLINE, Google Scholar, Wanfang, and China National Knowledge Infrastructure database to identify relevant reports about PPF using the following keywords: PPF, pancreaticothoracic fistula, pancreatic pleural fistula, and pancreatic pleural effusion. The references of the relevant studies were manually searched to identify additional relevant reports. Inclusion was limited to cases reported in pediatric patients published in the English and Chinese language between January 1976 and December 2018.

## 4 Results

The literature search identified 21 relevant reports, ^[3–23]^ and 17 of which were in English. A total of 33 pediatric patients were reported, of which 9 patients were insufficient for inclusion because of missing critical clinical data. Finally, a total of 25 pediatric patients including 1 patient from our hospital were included for review. Demographic, clinical characteristics and outcomes of pediatric patients are summarized in Table 1.

**Table 1:**
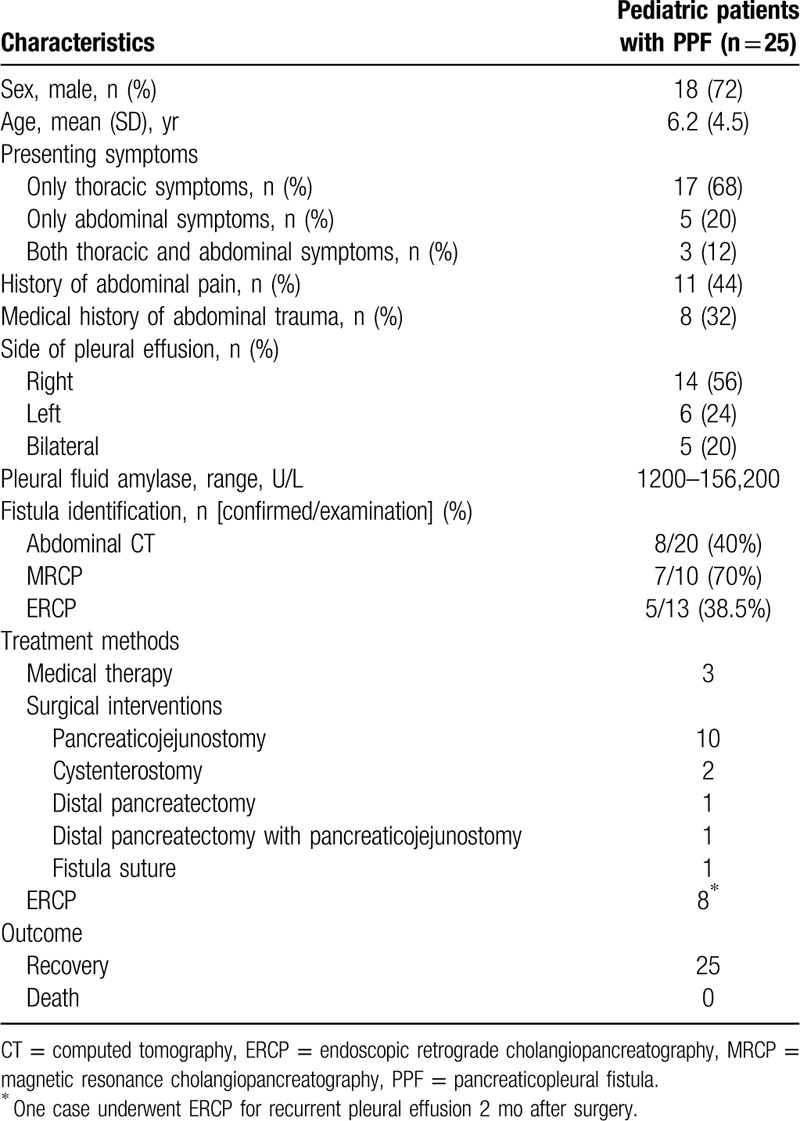
Demographics, clinical characteristics and outcomes of pediatric patients.

**Figure 6:**
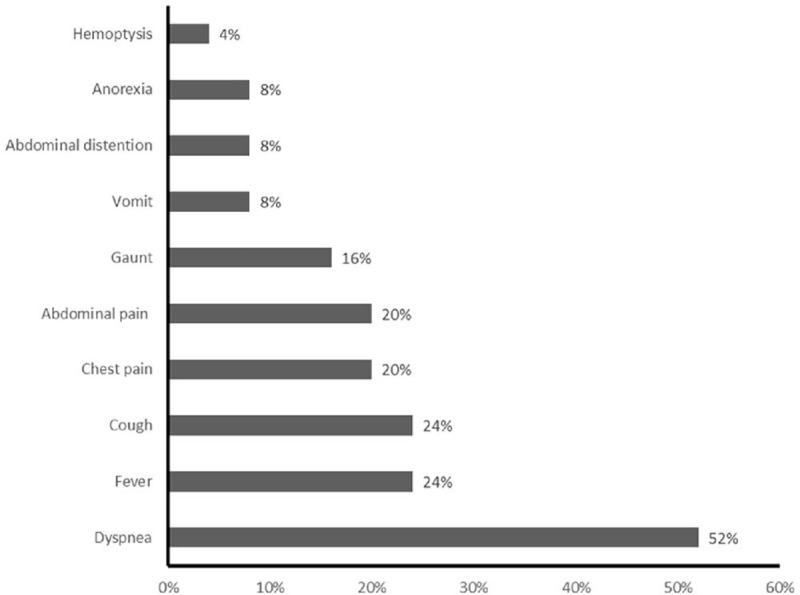
Presenting symptoms of 25 previously published pediatric patients with PPF. PPF = pancreaticopleural fistula.

Overall, most patients (68%) merely had thoracic symptoms, 5 patients (20%) only had abdominal symptoms, and others had both thoracic and abdominal symptoms. The most common complaint was dyspnea in 13 patients (52%). Other common presenting complaints included fever (n = 6, 24%), cough (n = 6, 24%), chest pain (n = 5, 20%), and abdominal pain (n = 5, 20%) (Fig. 6). Although most patients presented with thoracic symptoms, 11 patients (44%) had history of abdominal pain among those presented without abdominal pain. Furthermore, 8 patients (32%) had medical history of abdominal trauma, which were considered to be related to the onset of presenting symptoms. The most common etiology for pancreatic disorders was idiopathic (n = 15, 60%), followed by abdominal trauma (n = 8, 32%), and pancreatic divisum (n = 2, 8%). Increased pleural effusion amylase activity was observed in all patients, ranging from 1,200 to 156,200 U/L.

The most common imaging examination was abdominal CT, with fistula identification in 40% patients. Alternatively, 10 patients had MRCP, leading to a definitive diagnosis in 7 patients (70%). Diagnostic ERCP was performed in 13 patients, which was positive in only 5 patients (38.5%). Pancreatic fistula was not demonstrated in 8 patients (32%) using above imaging modalities. A total of 6 patients were diagnosed as the fistula was detected during surgical exploration. The remaining patients were diagnosed as PPF based on increased pleural effusion amylase level.

All patients were treated with medical therapy initially, including fasting, thoracentesis, proton pump inhibitor, somatostatin, antibiotic, and parenteral nutrition. This strategy was successful in only 3 patients (12%). Surgical intervention was performed in 15 patients, and was successful in 14 patients (93.3%). Finally, 8 patients underwent ERCP, including 1 had recurrent pleural effusion 2 months after surgery. This strategy was successful each time. Fortunately, there were no complications or deaths following surgical and endoscopic interventions.

## 5 Discussion

PPF occurs as a rare but serious complication of pancreatic disorders, such as acute pancreatitis, chronic pancreatitis, pancreatic pseudocyst, and pancreatic trauma. The precise incidence of PPF is still unclear, but it is reported to occur in approximately 0.4% of patients with acute or chronic pancreatitis, and 4.5% of patients with pancreatic pseudocyst.^[2]^ PPF occurs mostly among adult patients, on rare occasions, it can been seen in pediatric patients. We reviewed the literature on PPF published since 1976, which only yielded 33 pediatric patients. On the contrary, a total of more than 300 adult patients have been reported.^[24–26]^

The underlying mechanism of PPF involves disruption of pancreatic pseudocyst or pancreatic duct. Only when the disruption occurs posteriorly, pancreatic secretions can enter the retroperitoneal space, dissect via the aortic or esophageal diaphragmatic orifices into the mediastinum, and subsequently rupture into the pleural space to form PPF.^[27]^ PPF typically presents as massive pleural effusion. It rapidly accumulates and is refractory to therapeutic thoracentesis. It should be distinguished from the self-limiting pleural effusion which occurs in 3% to 17% of patients with acute pancreatitis, this type of reactive pleural effusion is often unilateral side, mild to moderate, and can resolve spontaneously during recovery.^[28]^

The principal clinical symptoms of PPF are pulmonary symptoms, which will cause a diagnostic dilemma as primary efforts are directed towards finding thoracic etiologies, thus resulting in a delay in the timely diagnosis. In our review, 2 thirds of pediatric patients only have thorax symptoms, namely dyspnea, cough, chest pain, and so on. The most prominent hallmark of PPF is the high amylase level. Amylase-rich pleural effusion can also been observed in non-pancreatic pathologies, such as tuberculosis, esophageal perforation, lymphoma, liver cirrhosis, or malignancy. However, only PPF-related pleural effusion has pancreatic-type amylase, while others have salivary-type amylase isoenzyme, which is produced by salivary glands, lung, or tumors.^[29]^Although there has been no established diagnostic threshold of pleural effusion amylase level for PPF, the amylase level of PPF is grossly elevated, usually above 1000 U/L, which is higher than other pathologies. And only PPF induced pleural effusion amylase level can exceed 50,000 U/L.^[29]^ In our review, the increased amylase level was detected in all pediatric patients, all were above 1000 U/L, and the maximum level is 156,200 U/L. As the underlying pancreatic disorders may be asymptomatic, pleural effusion amylase level should be evaluated in any cases of recurrent pleural effusion with unknown etiology, and the significantly elevated amylase level should cause a high index of suspicion for PPF.

Imaging examination is essential for diagnosing PPF. Owing to bowel gas artefact and inadequate respiratory cooperation especially in pediatric patients, transabdominal ultrasound has limit effectiveness for diagnosing PPF.^[30]^ Abdominal CT, MRCP and ERCP are commonly used imaging modalities for assessment of pancreatic fistula. Abdominal CT helps in demonstrating pancreatic parenchymal atrophy, pseudocyst, calcification, and duct dilatation. However, the sensitivity of abdominal CT for detecting PPF is low. King et al reported that abdominal CT can only identify fistula in 33% of cases.^[26]^ ERCP can more precisely demonstrate the pancreatic duct anatomy and identify the site of disruption. However, the positive rate of ERCP for identifying PPF is highly varied, as it is dependent on examination timing, anatomical variations, and success rate of cannulation. In addition, it is unable to clearly visualize a fistula if the site of ductal rupture occurs distal to the site of ductal stricture or even obstruction. King et al reported that ERCP is helpful in the diagnosis of PPF in 78% of the cases.^[26]^ While Nordback et al reported 5 cases with suspected PPF, none of the 5 cases could the fistula be visualized under ERCP, as the main pancreatic duct was completely obstructed in the head of the pancreas in all patients.^[31]^ Furthermore, ERCP is an invasive interventional procedure, with risk of potentially life-threatening complications. As for pediatric patients, radiological protection of gonad remains an important issue. Therefore, the use of ERCP as a first-line diagnosed method for PPF is discouraged. MRCP is a noninvasive imaging modality, with the ability of demonstrating pancreatic duct anatomy upstream to the site of ductal stricture or obstruction. Ali et al reported that MRCP was helpful in the diagnosis of PPF in 80% of cases, while ERCP and CT scan were useful in 78% and 47% respectively.^[25]^ In our review, the sensitivity of MRCP (70%) for diagnosing PPF is higher than ERCP (38.5%) and abdominal CT (40%). Considering it is a non-invasive, radiation-free assessment modality with preferable diagnosis capability, we recommend MRCP as the first choice for diagnosing PPF.

There is no established treatment algorithm for PPF among pediatric patients, and the current evidence for the management of PPF is limited to case reports and mainly for adult patients. Traditionally, medical therapy is initially attempted for 2 to 3 weeks, followed by endoscopic or surgical interventions for those who failed conservative treatment.^[29]^ Medical therapy, especially somatostatin can significantly reduce pancreatic exocrine secretions so as to hasten the closure of ductal disruption.^[32]^ However, the success rate of medical therapy alone varied, ranging from 31% to 65%.^[29]^ King et al reported that the success rate of medical therapy is 31%, obviously lower than surgical interventions (94%).^[26]^ An inappropriate prolonged period of medical therapy may delay the resolution of the fistula and prolong the duration of therapy. In our review, only 3 patients (12.5%) recoved after medical therapy. Surgical intervention is an important therapeutic option for PPF before the era of therapeutic endoscopy. The surgical methods are highly variable, mostly depending on ductal anatomy. For those with proximal lesions, pancreaticojejunostomy or cystenterostomy is recommended; while for those with distal lesions, distal pancreatectomy with or without pancreaticojejunostomy is recommended.^[25]^ Takeda et al reported a 100% success rate of surgical therapy for PPF among 9 pediatric patients.^[19]^ In our review, 15 pediatric patients underwent surgical interventions after failed medical therapy, this strategy was successful in all patients except 1 had recurrence 2 months later requiring endoscopic interventions. ERCP with pancreatic duct stenting so as to restore the anatomic continuity of pancreatic duct is an effective therapeutic option associated with minimal morbidity and mortality. Ideally, endoscopic stenting should bridge the site of disruption.^[33]^ It can not only decreases the pancreatic duct pressure, but also play an important role in sealing the disruption. Pai et al reported a 96.4% success rate of endoscopic therapy in the treatment of internal pancreatic fistulas, including 13 PPFs, although no leakage was found in 28.6% cases during ERCP.^[34]^ In our review, ERCP was performed in 8 pediatric patients, leading to resolution of the fistula in each case. For our case, no leakage was found during ERCP, meanwhile minor papilla cannulation and dorsal ductography were failed. We suggested that NPD placement would create a free pathway for pancreatic secretions to flow into the duodenum so as to achieve decompression of the pancreatic duct and hasten the closure of the fistula. After placement of NPD, the decreased chest tube drainage volume and no recurrence during recovery confirmed the effectiveness of endoscopic intervention. However, it is noteworthy that ERCP is technically demanding and requires substantial experience to avoid potentially life-threatening complications especially in pediatric patients. Therefore, careful selection of patients is essential. ERCP is only recommended for those with ductal stricture, ductal disruption in the head and body of the pancreas or failed medical therapy. While complete ductal obstruction or failed endoscopic therapy favor surgical intervention.

## 6 Conclusions

PPF is a rare disease and requires a high index of suspicion in pediatric patients presenting with recurrent massive pleural effusion. A thoughtful inquiry of medical history may be helpful. After detailed laboratory examination, MRCP is the first-choice imaging modality for diagnosing PPF. Management strategies should be tailored to the pancreatic duct morphology. ERCP is recommended when presence of ductal stricture or when medical therapy fails, while surgical intervention should be reserved for patients with failed ERCP or complete ductal obstruction.

## Author contributions

**Conceptualization:** Jing Yang, Xiao-feng Zhang

**Data curation:** Jing Yang, Lei Lu, Hang-bin Jin

**Formal analysis:** Jing Yang, Jian-feng Yang

**Writing – original draft:** Jing Yang, Lei Lu

**Writing – review & editing:** Jing Yang, Jian-feng Yang, Xiao-feng Zhang

## Supplementary Material

Supplemental Digital Content
