# Supplementary material for: Endoscopic management of pancreaticopleural fistula in a pediatric patient: A case report and literature review
Source: Medicine (Baltimore). 2020 Jun 5;99(23):e20657. doi: 10.1097/MD.0000000000020657 (PMC7306390; doi:10.1097/MD.0000000000020657)
Supplement: Supplemental Digital Content [file medi-99-e20657-s001.docx]

**Timeline Picture of Patient’s History**

**Relevant Past Medical History:** Sporadic epigastric pain during the last year before hospitalization.

**Diagnostic Evaluations:**

Chest X-ray: Massive pleural effusion in the right thorax.

Abdominal CT: Dilated irregular pancreatic duct.

MRCP: Dilated pancreatic duct and a fistulous tract.

**Current Illness:** Cough and dyspnea for one month.

**Physical examination**: Tachypnea, decreased breath sounds and dull percussion note on the right thorax.

**Diagnosis:** Chronic pancreatitis with pancreaticopleural fistula

**Final Follow-up:** Healthy and symptom-free.

**Initial Treatment and Referrals:** After failed medical therapy, ERCP was performed.

**December,**

**2016**

**December,**

**2018**

**Resolution of this Episode of Care**

**November,**

**2016**
